# Falls in young, middle-aged and older community dwelling adults: perceived cause, environmental factors and injury

**DOI:** 10.1186/1471-2458-5-86

**Published:** 2005-08-18

**Authors:** Laura A Talbot, Robin J Musiol, Erica K Witham, E Jeffery Metter

**Affiliations:** 1 Graduate School of Nursing, Uniformed Services University of the Health Sciences, 1335 East West Highway, Silver Spring, Maryland 20910, USA; 2 Holy Cross Hospital, 1500 Forrest Glen Road, Silver Spring, Maryland 20910, USA; 3 The Henry M. Jackson Foundation, 1401 Rockville Pike, Suite 600, Rockville, Maryland 20852, USA; 4 Intramural Research Program, National Institute on Aging, National Institutes of Health, Harbor Hospital, 3001 South Hanover Street, Baltimore, Maryland 21225, USA

## Abstract

**Background:**

Falls in older people have been characterized extensively in the literature, however little has been reported regarding falls in middle-aged and younger adults. The objective of this paper is to describe the perceived cause, environmental influences and resultant injuries of falls in 1497 young (20–45 years), middle-aged (46–65 years) and older (> 65 years) men and women from the Baltimore Longitudinal Study on Aging.

**Methods:**

A descriptive study where participants completed a fall history questionnaire describing the circumstances surrounding falls in the previous two years.

**Results:**

The reporting of falls increased with age from 18% in young, to 21% in middle-aged and 35% in older adults, with higher rates in women than men. Ambulation was cited as the cause of the fall most frequently in all gender and age groups. Our population reported a higher percentage of injuries (70.5%) than previous studies. The young group reported injuries most frequently to wrist/hand, knees and ankles; the middle-aged to their knees and the older group to their head and knees. Women reported a higher percentage of injuries in all age groups.

**Conclusion:**

This is the first study to compare falls in young, middle and older aged men and women. Significant differences were found between the three age groups with respect to number of falls, activities engaged in prior to falling, perceived causes of the fall and where they fell.

## Background

Falls are a health problem in need of preventive intervention and research [1] with prior studies focused on older adults [2]. In older people, falls are a risk factor for disability and frailty, and exacerbate the disablement process [3,4]. Numerous studies have reported on the circumstances surrounding falls in older adults including descriptive studies detailing falls causation, related injuries, and risk factor determination [5,6]. This information on falls has also led to the development and testing of various preventive interventions for older adults including preventive screening for those at risk, strength and balance training, home hazard assessments with modifications, and Tai Chi [5]. In contrast to studies in older people, little attention is given to falls in young and middle-aged adults where falls represent a risk for injury with related expenses and potential interference with work and family [7,8]. Publications are lacking that describe the incidence, prevalence and circumstances surrounding falls in these age groups. This descriptive study examines and compares falls in young, middle and older aged men and women. The focus is on the faller's activity, environment, and perception as to what caused the fall.

## Methods

### Sample

Participants included 1497 volunteers from the Baltimore Longitudinal Study of Aging (BLSA), a prospective study of the National Institute on Aging, initiated in 1958 as an open panel cohort of participants recruited primarily from the Baltimore-Washington, DC area. Participants return for evaluation at one to five year intervals for 1–3 days of physiological and psychological tests at the Gerontology Research Center (GRC). BLSA participants are community residing, predominantly upper middle class and health conscious. A falls questionnaire was completed by 757 men and 740 women aged 20 to 92 years from 1996 to 2001 with 96.5% scoring < 7 on the Blessed Information-Memory-Concentration Test [9] and none having a diagnosis of dementia at that visit. The young age group (20–45 years) included 292 participants, the middle aged group (46–65 years) had 616 participants and the older aged group (> 65 years) included 589 participants.

### Descriptive variables

Physical activity was based on a self-report of 97 activities performed over the previous 2 years and converted into metabolic equivalents of resting oxygen consumption (MET-minutes) per 24 hours (see Talbot et. al. [10] for details). Body mass index (BMI) was calculated from weight in kilograms divided by height in meters squared. The Center for Epidemiologic Study Depression (CES-D) Scale, a validated self-report instrument, was used to measure depressive symptoms [11,12].

### Instruments

The History of Falls questionnaire is a 17-item survey developed in 1996 by Dr. Ann Myers based on an extensive literature review, a panel of experts, and her research on falls [13]. The questionnaire queried the circumstances surrounding a fall, specifically, activities prior to falling, perceived cause, environmental factors, and a description of injuries. The participant could check multiple responses for contributing environmental hazards and their perceived cause for falling. A specific definition of a fall was not used in this questionnaire.

For these analyses, participants were classified as fallers if they had at least one fall in the past two years. Only one fall per faller (the most serious fall) was used in the analyses. If injured in the fall, participants were then prompted with a list of possible injuries.

*Activities prior to falling *were categorized according to type of activity: (1) Ambulation – included walking, turning and standing; (2) Transferring – included getting in/out of bed, chair, wheelchair or car, getting on/off toilet, climbing stool, chair, or ladder, and falling off stool or chair; (3) Running; (4) Sports – included exercising, dancing, cycling; (5) Stairs/curb; and (6) Other – such as reaching overhead and bending down.

*Perceived causes *of falling were grouped into: 1) accident/environment related – such as slipped, missed seat, was bumped/pushed, slid off surface, bumped into object, or furniture/equipment broke; 2) collapse episode – included passing out or legs gave way; 3) dizziness/vertigo/weakness – included dizziness, light-headed, weakness or paralysis; 4) balance/gait impairment – included tripped/stumbled, quick movement, or lost balance; and 5) other – which contained causes not listed on the questionnaire and circumstances where the faller was uncertain of the cause.

*Environmental factors *were grouped into: 1) wet surface/slippery footwear; 2) uneven surface/steps – includes steps of unequal height, and indistinguishable surface colors; 3) objects on surface/rug – includes tripped/slid on rug; 4) external forces – furniture/stair/step/railing broke and something moved or bumped the participant; 5) icy surface; and 6) other – which included eyeglasses, darkness/dimness or environment, glare, "other", or "uncertain". Participants were given three choices as to where the fall occurred: 1) inside the home, 2) inside a building but not at home, or 3) outside.

*Injuries sustained from a fall *were grouped into four treatment categories: fractures, treated injury, untreated injury, and no injury. For these analyses, the most serious injury reported was included. Treated injury was any injury that caused the participant to see a physician, and/or go to the emergency room or hospital. Fractures, though treated, were categorized separately from treated injury. An untreated injury was any injury caused by a fall where the participant did not see a physician, or go to the emergency room or hospital. The no injury category included participants who reported falling but did not report injuring themselves as a result of the fall. This categorization of injury has been used previously [14] and represents the severity of the injury as initially perceived by the participant.

## Procedure

The History of Falls questionnaire was mailed to the participants and completed prior to visiting the GRC. A nurse practitioner or physician's assistant reviewed the questionnaire with the participant for accuracy. The study was approved by the Institutional Review Board at Johns Hopkins Bayview Medical Center, and all participants gave signed, informed consent.

### Data analysis

Descriptive statistics of mean, standard deviation, percentages and frequencies were calculated for all variables. The Pearson chi-square test was used to examine associations between gender and categorical variables related to falling. Analysis of variance was used to determine activity differences between fallers for the three age groups. The proportion of injuries sustained from falling was grouped according to the severity of the injury. The significance was set at the 0.05 level. Statistical analyses were performed using SPSS (version 10.1).

## Results

Participant characteristics are shown in Table 1. Participants are generally well educated, Caucasian, married, overweight (BMI > 25), screened negative for depression and report being in good to excellent health. Young fallers were more active than older fallers > 65 years (F(2,288) = 3.16, p > 0.04).

**Table 1:** Sample characteristics of the population

	**Total Population ** (N = 1497)	**Young (20–45 years) ** (N = 292)		**Middle (46–65 years) ** (N = 616)		**Old (> 65 years) **(N = 589)	
		Fallers (N = 54)	Non-Fallers (N = 238)	Fallers (N = 131)	Non-Fallers (N = 485)	Fallers (N = 205)	Non-Fallers (N = 384)

**Age **(years)	59.5 ± 16.5	34.0 ± 6.9	35.6 ± 6.8	55.9 ± 5.5	55.1 ± 5.6	78.2 ± 7.1	75.2 ± 6.7
**Gender ** (% male)	50.6% (757)	46.2% (23)	47.9% (114)	31.3% (41)	44.4% (220)	51.7% (106)	65.9% (253)
**Race ** (% Caucasian)	72.1% (962)	65.2% (30)	56.2% (109)	69.2% (83)	66.2% (286)	88.4% (167)	81.3% (287)
							
**Marital Status **(% subj.)							
Single (Single, Divorced)	23.5% (299)	37.5% (15)	31.5% (58)	27.7% (31)	30.7% (126)	14.4% (27)	12.3% (42)
Married	66% (842)	62.5% (25)	62.5% (115)	67.0% (75)	67.2% (276)	62.0% (116)	68.9% (235)
Widowed	10.5% (134)	NONE	6.0% (11)	5.4% (6)	2.2% (9)	23.5% (44)	18.8% (64)
							
**Annual Income**							
(% > $65,000)	57.3% (718)	57.5% (23)	54.9% (100)	61.8% (68)	66.3% (268)	44.2% (80)	53.4% (176)
							
**College Graduates **(%)	75.9% (933)	84.2% (32)	80.1% (141)	76.2% (80)	75.3% (298)	71.0% (130)	76.1% (252)
							
**Body Mass Index ** (kg*m^-2^)							
Male	27.6 ± 3.8 (739)	27.9 ± 4.1 (21)	27.5 ± 3.7 (113)	28.0 ± 4.5 (41)	27.8 ± 3.6 (219)	27.1 ± 3.6 (100)	27.7 ± 4.1 (245)
Female	26.3 ± 4.9 (685)	24.2 ± 3.6 (30)	24.9 ± 4.9 (120)	27.6 ± 5.2 (82)	27.0 ± 5.4 (246)	25.7 ± 3.9 (92)	26.0 ± 4.6 (115)
							
**CES-D Depression Score**^1^							
(% > 15)	9.5% (136)	18.5% (10)	12.2% (28)	3.3% (4)	7.9% (37)	11.6% (21)	9.7% (36)
							
**Self-Reported Health**							
(% Excellent or Good)	93.7% (1152)	100.0% (41)	96.7% (175)	90.8% (99)	98.5% (391)	84.0% (152)	91.9% (294)
							
**Activity Level**							
(MET-minutes/24 hours)	2228 ± 660 (1092)	2403 ± 528 (36)	2339 ± 521 (172)	2218 ± 492 (95)	2163 ± 487 (356)	2160 ± 546 (160)	2262 ± 977 (273)

Values are mean ± SD except where indicated

^1^CES-D = Center for Epidemiologic Study Depression Scale; Score > 15 indicates significant depressive symptoms

Twenty-six percent of the participants fell in the previous two years (390 fallers of 1497 participants). One or more falls occurred in 18.5% of young adults (54 fallers of 292 young participants), 21% of middle-aged adults (131 fallers of 616 middle-aged participants) and 35% of older adults (205 fallers of 589 older participants). Significant differences were found between women and men (χ^2 ^= 10.27; p < 0.001) and between age groups ((χ^2 ^= 39.41; p < 0.001). Older men and women were more likely to report a fall in the previous two years than middle-aged and young men and women.

**Figure 1:**
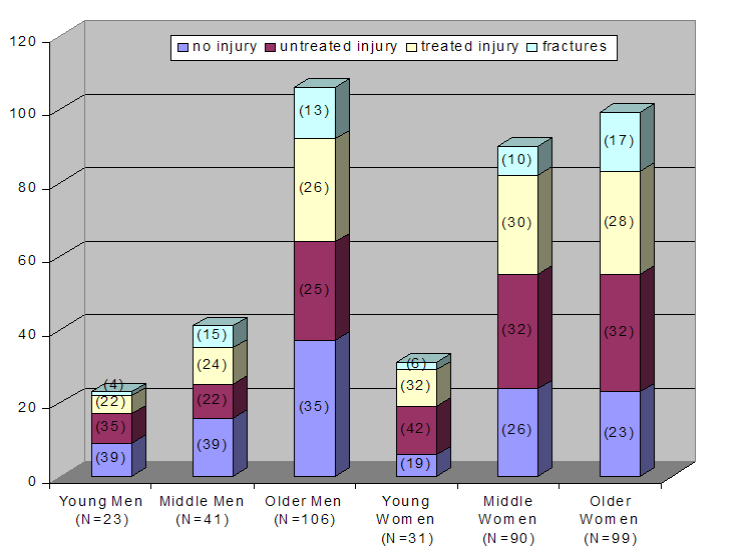
Frequency comparison of injury groups in fallers by age and gender. The X-axis displays the breakdown of all fallers into age and gender groups. The Y-axis shows the total number of participants. Percentages of each injury type per age/gender groups are displayed parenthetically on the graph bars.

Table 2 details the responses to the questionnaire in relation to their most serious fall. The activity most frequently cited as causing the fall was ambulation in all age-groups and both genders. Younger participants tended to fall more with running, older participants while walking. The young participants fell more outdoors, while the percentage of falls indoors increased from the middle to the older age group. Participants' perceived the cause of the fall to be balance/gait impairment and accidents/environment related. From the middle to old age group, the reporting of accidents/environment related falls declined while balance/gait impairment increased. Figure 1 illustrates the severity of the most serious injury suffered (by treatment groups) based on age group and gender.

**Table 2:** Factors preceding the most serious fall

	**Total Cohort**			**Men**			**Women**		
	**Young** **(N = 54)**	**Middle** **(N = 131)**	**Old** **(N = 205)**	**Young** **(N = 23)**	**Middle** **(N = 41)**	**Old** **(N = 106)**	**Young** **(N = 31)**	**Middle** **(N = 90)**	**Old** **(N = 99)**

ACTIVITIES PERFORMING PRIOR TO THE FALL*									
Ambulation	31.5(17)	45(58)	56.5(113)	13(3)	42.5(17)	58.3(60)	45.2(14)	46.1(41)	54.6(53)
Transferring	9.3(5)	10.9(14)	9(18)	8.7(2)	17.5(7)	8.7(9)	9.7(3)	7.9(7)	9.3(9)
Running	20.4(11)	6.2(8)	5.5(11)	21.7(5)	5(2)	4.9(5)	19.4(6)	6.7(6)	6.2(6)
Sports/exercise/dancing/ bicycling	22.2(12)	13.2(17)	6.5(13)	39.1(9)	10(4)	10.7(11)	9.7(3)	14.6(13)	2.1(2)
Stairs/curb	7.4(4)	11.6(15)	9(18)	13(3)	12.5(5)	7.8(8)	3.2(1)	11.2(10)	10.3(10)
Other^1^	9.3(5)	13.2(17)	13.5(27)	4.3(1)	12.5(5)	9.7(10)	12.9(4)	13.5(12)	17.5(17)

PERCEIVED CAUSE OF THE FALL **									
Accident/Environment	37(20)	30.2(38)	15.8(32)	39.1(9)	32.5(13)	16.2(17)	35.5(11)	29.1(25)	15.5(15)
Collapse Episode	5.6(3)	6.3(8)	3.5(7)	13(3)	10(4)	4.8(5)		4.7(4)	2.1(2)
Dizziness/Vertigo/Weakness	5.6(3)	1.6(2)	2.5(5)			1(1)	9.7(3)	2.3(2)	4.1(4)
Balance/Gait Impairment	38.9(21)	49.2(62)	61.9(125)	39.1(9)	47.5(19)	62.9(66)	38.7(12)	50(43)	60.8(59)
Other/Uncertain^2^	13(7)	12.7(16)	16.3(33)	8.7(2)	10(4)	15.2(16)	16.1(5)	14(12)	17.5(17)

ENVIRONMENTAL FACTORS LEADING TO THE FALL***									
Wet surface/slippery footwear	9.3(4)	17(16)	9.5(14)	11.1(2)	13.8(4)	7.6(6)	8(2)	18.5(12)	11.6(8)
Uneven surface/steps	20.9(9)	25.5(24)	27(40)	16.7(3)	24.1(7)	32.9(26)	24(6)	26.2(17)	20.3(14)
Objects on surface/rug	9.3(4)	7.4(7)	16.2(24)	5.6(1)	6.9(2)	15.2(12)	12(3)	7.7(5)	17.4(12)
External forces	7(3)	4.3(4)	2(3)	5.6(1)	6.9(2)	2.5(2)	8(2)	3.1(2)	1.4(1)
Icy surface	20.9(9)	12.8(12)	14.2(21)	22.2(4)	24.1(7)	13.9(11)	20(5)	7.7(5)	14.5(10)
Other^3^	32.6(14)	33(31)	31.1(46)	38.9(7)	24.1(7)	27.8(22)	28(7)	36.9(24)	34.8(24)

Values are percentages (frequency)

*Activities Performing Prior to The Fall (Total Cohort: χ^2 ^= 31.01; p < 0.001; Men:χ^2 ^= 31.51; p < 0.001; Women: χ^2 ^= 17.44; p > 0.05).

**Perceived Cause (Total Cohort:χ^2 ^= 29.03; p < .001; Men: χ^2 ^= 15.61; p < 0.05; Women: χ^2 ^= 15.54; p < 0.05).

***Environmental Factors (Total Cohort:χ^2 ^= 11.52; p > 0.05; Men: χ^2 ^= 8.04; p > 0.05; Women: χ^2 ^= 10.34; p > 0.05).

^1^Other represents responses of "reaching overhead", "bending down", "uncertain", or "other"

^2^Other/Uncertain represents responses of "other" or "uncertain" as to the cause of the fall.

^3^Other represents responses of "new eyeglasses" and/or "darkness/dimness of environment" and/or "glare from lights"

Most frequently, the young group injured their wrist/hand, knees and ankles; the middle-aged injured their knees; and the older group injured their head and knees. Of those who fell, there were no differences in the percentage of injuries across age groups for men and women (Men χ^2 ^= 0.30; p = 0.86; Women: χ^2 ^= 0.75; p = 0.69). Women reported a higher percentage of injuries and women in the youngest age group reporting more injuries (80.6% or 25 of 31 fallers) than any other group. Table 3 displays frequencies of falls and most serious injury type.

**Table 3:** Frequency of falls and injuries* in fallers by age group and gender over two years

	**Total Cohort**			**Men**			**Women**		
	**Young** **(N = 292)**	**Middle** **(N = 616)**	**Old** **(N = 589)**	**Young** **(N = 137)**	**Middle** **(N = 261)**	**Old** **(N = 359)**	**Young** **(N = 155)**	**Middle** **(N = 355)**	**Old** **(N = 230)**

**Total Number of Falls (percentages)**									
0	238(81.5)	485(78.7)	384(65.2)	114(83.2)	220(84.2)	253(70.5)	124(80.0)	265(74.5)	131(57)
1	25(8.6)	74(12.0)	99(16.8)	12(8.8)	22(8.4)	50(13.9)	13(8.4)	52(14.6)	49(21.3)
2	12(4.1)	24(3.9)	53(9.0)	3(2.2)	10(3.8)	31(8.6)	9(5.8)	14(3.9)	22(9.6)
3	3(1.0)	9(1.5)	25(4.2)	0	2(0.8)	12(3.3)	3(1.9)	7(2.0)	13(5.7)
4	3(1.0)	11(1.8)	16(2.7)	1(0.7)	2(0.8)	8(2.2)	2(1.3)	9(2.5)	8(3.5)
5+	11(3.8)	13(2.1)	12(2.0)	7(5.1)	5(1.9)	5(1.4)	4(2.6)	8(2.2)	7(3.0)
Total Fallers	54(18.5)	131(21.3)	205(34.8)	23(16.8)	41(15.7)	106(29.5)	31(20.0)	90(25.3)	99(43.0)

**If Fell, Injured?**									
No	15(27.8)	40(30.5)	60(29.3)	9(39.1)	16(39.0)	37(34.9)	6(19.4)	24(26.7)	23(23.2)
Yes	39(72.2)	91(69.5)	145(70.7)	14(60.9)	25(61.0)	69(65.1)	25(80.6)	66(73.3)	76(76.8)

**Most Serious Injury Sustained from the Fall (percent of injured fallers)**									
Cut/Laceration^1^	6(15.4)	11(12.1)	29(20.0)	1(7.1)	2(8.0)	17(24.6)	5(20.0)	9(13.6)	12(15.8)
Bruise/Hematoma^2^	11(28.2)	25(27.5)	40(27.6)	4(28.6)	7(28.0)	14(20.2)	7(28.0)	18(27.3)	26(34.2)
Sprain or Strain^3^	18(46.2)	25(27.5)	19(13.1)	8(57.1)	7(28.0)	12(17.4)	10(40.0)	18(27.3)	7(9.2)
Fracture	3(7.7)	15(16.5)	31(21.3)	1(7.1)	6(24.0)	14(20.3)	2(8.0)	9(13.6)	17(22.4)
Other^4+^	1(2.6)	15(16.5)	26(17.9)	0	3(12.0)	12(17.4)	1(4.0)	12(18.2)	14(18.4)

Values are frequencies (percentages)

*most serious injury sustained only

1 Out of all Cut/Lacerations, 34 were Untreated and 12 were Treated Injuries

2 Out of all Bruise/Hematomas, 51 were Untreated and 25 were Treated Injuries

3 Out of all Sprain/Strains, 26 were Untreated and 36 were Treated Injuries

4 Out of all Other Injuries, 7 were Untreated and 35 were Treated Injuries

^+^Other also includes responses of "burn" and not reported

## Discussion

This is the first study to compare falls in young, middle and older community-dwelling men and women. The main finding is the high frequency of fallers in all age groups and the frequency of injury. We found significant differences between the three age groups with respect to number of fallers, activities leading to falling, perceived cause and location of the fall. Injuries across the age groups and between genders were not significantly different. Women had a higher percentage of fallers than men regardless of age group consistent with both retrospective [15,16] and prospective studies [17].

### Activities causing the fall

Significant age differences were found in activities leading to falling among men (p < 0.001), but not women (p = 0.07) (Table 2). The highest percentage of individuals fell while ambulating regardless of age group with young people falling while participating in sports, exercise or running. Also, vigorous activities contributed to falls in all groups except older women. Our results contrasted with Tideiksaar [18] where the majority of community-dwelling older aged persons (average age of 84 years) fell while transferring (43%) and walking (28%).

In older fallers [19], the majority of falls occurred while walking on a level surface during ordinary daily activities in the absence of hazardous behavior. In a Japanese study, falls occurred most frequently while walking [20]. Speechley and Tinetti [21] found in a one year follow up of community dwellers over 75 years that active individuals were more likely to fall during displacing activities (climbing ladders, engaged in sports, or on stairs) while the frail fell during routine non-displacing daily activities.

Although these comparison studies are of older adults, the functional status varies greatly in such a broad age group. This has led some researchers to compare their results by factors of functional status and/or independence [21,22]. Koski et al. [22] found that while major injuries in the independent older persons were more likely to be a result of running or jumping activities, no activity was related to falls resulting in a major injury in the less functionally independent older people in their sample. This difference in risk factors for falling and being injured in older persons by functional status further complicates comparing studies when there is no or different means to identifying functional status of the study sample.

### Perceived causes

Most older fallers reported tripping as the cause for their fall [18,23], with a large percentage unable to give any reason in a one year retrospective study [24]. No information was found comparing young, middle and older fallers' perceived cause of their fall.

In the present study, of those that fell, age group differences were found for their perceived cause of falling (table 3). Gait and balance problems were the most frequently cited cause in all age and gender groups. Accidents/environment reasons were cited more frequently in the young (37%; 20 of 54 fallers) and the middle-aged groups (30%; 38 of 126 fallers) compared to older fallers (15.8%; 32 of 202 fallers).

### Environmental factors

As people age, changes in balance, proprioception, muscle strength, attention and vision make compensation to environmental hazards more difficult [25-27]. Environmental hazards are more likely to precipitate the fall in healthy active older individuals than in their frailer counterparts [21,28]. In a Swedish hospital-based study, slipping on ice or snow caused 3.5 injuries per 1000; older women and young men (20–29 years) had the highest incidence of injury with half being fractures [29]. A study of hospital-treated fractures in Norway revealed 90% of fractures in adults over 65 were caused by falls; the fracture risk increased fivefold in months with ice and snow [30]. No other studies were found that examined environmental factors and falls in younger and middle-aged adults, other than studies regarding specific occupational hazards. In our study, the environmental factor contributing to the highest percentage of falls was uneven surfaces/steps (Table 2).

### Where fall occurred

Older fallers are more likely to fall indoors than outdoors [24]. The very old (average age 84 years) in Tideiksaar's study [18] fell most often at home (81%). Campbell et al [17] found in a prospective study of participants (over 69 years) from a rural setting no gender difference in fall rate; however men were more likely to fall outside and at more intense levels of activity. A Danish study of older adults using a 24-hour recall method found that one specific component of the home environment had a strong association with falls; vinyl or linoleum on the floors [15]. Active participants tend to fall outside the home and the frail older participants fell inside the home [31]. There was a significant difference in our population with regard to where participants fell by age group (χ^2 ^= 19.699; p < .001). The highest percentage fell outside (62.5%: 244 of 390 fallers). Less than 4% (2 of 54 fallers) of the younger group in our study reported falling in their own home. This compares to the middle-aged and older groups where 17.6% (23 of 131 fallers) and 29.3% (60 of 205 fallers) fell in their homes respectively.

With regard to gender, the percentage of falls occurring inside the home increased with age. Thirty-seven percent (36 of 97 women fallers) of the older women and 23% (24 of 106 men fallers) of the older men reported that they fell inside their own home.

### Injury severity

Tinetti et al [32] found in a prospective study that 24% of the falls in community dwelling older aged persons resulted in serious injury and 6 percent had fractures. Gryfe et al [33] found 17.5% of falls in older aged persons living in an apartment setting suffered serious injury (6.1% fractures and 11.4% soft tissue injury). In the Netherlands, community dwellers (> 70 years) who fell within one year previous to a postal survey, 8% reported a major injury and 49% had minor injuries [31]. While in another study (mean age = 84), 18% of fallers sustained an injury, and 14% of the injuries were fractures [18].

In a trauma study, a 6% fall injury rate was found for the frail older people contrasted to a 0.4% rate for 40 year olds [34]. In a trauma registry study in Connecticut, serious injury was seen in 32% of participants > 65 years of age and only 11% for the younger group [1]. No information was found comparing type and severity of injury in young, middle and older age groups of fallers.

Our population reported a higher percentage (70.5%; 275 of 390 fallers) of injuries from their falls than previous prospective [32,33,35] and retrospective studies [20,31]. Part of the difference may relate to different definitions for categorizing the injury. For example, Tinetti et al [32] defined a serious injury as all nonvertebral fractures, injuries resulting in an emergency room stay > 24 hours, hospital admission, bed rest > 48 hours, or activity restriction > 72 hours. This reflects a more restrictive definition than used in our report, which would lead to lower rates. Koski et al [22] defined a major injury by fractures, joint dislocations, lacerations needing sutures, and intracranial injuries. This prospective study found that 32% of all fallers suffered a major injury. However, by their definition, major injury included fractures. Breaking down their major injury category into fractures and other major injuries, a fracture rate of 15.5% is revealed showing it be very similar to our results [22]. Tideiksaar [18] also reported a fracture rate that was comparable to the observations in this study.

Speechley and Tinetti [21] found 22% of falls by vigorous participants resulted in serious injuries compared to 6% of falls by frail participants, suggesting active older people are involved in activities that would cause greater injury if a fall occurred. This higher rate of serious injury in the active individuals could also be a result of increased exposure to risky activities and environments or also a higher speed and force of an activity leading to a fall. Koski et al [22] found among more active participants the primary activity connected to major injurious falls was running or jumping; however, the highest rate of major injury was in the more frail participants.

Previous studies have focused on older populations and not the entire adult lifespan as in this report. We found that severity of injury, as defined by treatment, across age and gender was not significantly different. The percentage of falls resulting in fractures increased from young (5.56%; 3 of 54 young fallers) to middle (11.5%; 15 of 131 fallers) to oldest (15.1%; 31 of 205 fallers) age groups. In the middle and older age groups, the occurrence of treated and untreated injuries resulting from falls was similar. A higher percentage of women reported any injury from the fall. Interestingly, younger women reported a higher percentage of injuries (80.6%; 25 of 31 fallers) than any other age group. Middle-aged men (14.6%; 6 of 25 injured fallers) joined older men (13.2%; 14 of 69 injured fallers) and older women (16.2%; 17 of 76 injured fallers) as the groups reporting the highest percentage of fractures.

### Anatomic site of injury

The most common injury sites for older (> 65) adults were face (17%), upper extremity (16%), hand/wrist (14%), and head (12%), with lower extremity and ankle/foot less common [36]. Another study of injury in older adults (51% were falls) reported legs (43%) or arms (39%) most frequently occurring [37].

No study has described common injury sites from a fall across the adult age span. Our study showed that the young group injured their wrist/hand most frequently (20%) while middle-aged adults injured their knees (19%) and older adults injured their head (15%).

### Study limitations

Study limitations include the cross-sectional nature of the design, the self-report of injury, and memory recall of falls spanning 2-years. In any study of falls, the incidence of self-reported falls is underestimated and less reliable than observed events with participants more likely to remember falls that resulted in injury. Also, we did not control for the number and type of chronic conditions that make the older population more susceptible to falls. The severity categories used here limit how well we are able to compare our results with other studies that utilized more restrictive criteria.

The BLSA represents a well educated and health conscious group of individuals who are more likely to practice health promoting behaviors and therefore more likely to remember, report falls, and seek medical help than the general population. As a result, one potential source of bias would be a higher rate of reported falls and subsequent injury than in studies with a more diversified population, thus limiting comparisons.

## Conclusion

Falling is a health risk meeting all criteria for prevention: high frequency, evidence of preventability and high burden of morbidity. While falls in young adults may be considered a lifestyle issue related to sports and vigorous activity, more attention may need to be directed to the middle-aged adult where approximately one in four reported falling at least once in a two year period. Falling often results from multiple concurrent problems including environmental and behavioral factors as well as disease processes. Middle-aged adults progressively start to show higher incidences of diseases and medication use, along with lower levels of physical activity, and physiological changes that begin to alter postural stability. Physical exercise and balance training have shown some benefit in older people, and may be of benefit in middle-age, but this has not been studied [38]. Events in this middle-aged group are likely to predispose individuals for the higher risks that lead to falls in later years.

## Competing interests

The author(s) declare that they have no competing interests.

## Authors' contributions

LAT and EJM were the key authors in the conception, design, coordination, and drafting of the manuscript as well as the analysis and interpretation of the data. RJM participated in the design and interpretation of the data and helped in drafting the manuscript. EKW contributed substantively by revising the manuscript critically for intellectual content and participating in the interpretation of data and the revision of the manuscript. All authors read and approved the final manuscript.

## Pre-publication history

The pre-publication history for this paper can be accessed here:


